# Is an aggressive surgical approach worthwhile in biliary cancer?

**DOI:** 10.1186/1477-7800-4-26

**Published:** 2007-10-23

**Authors:** Nadja Ristagno, Alexander Knuth, Bernhard C Pestalozzi

**Affiliations:** 1 Department of Oncology, University Hospital, Raemistrasse 100, 8091 Zürich, Switzerland

## Abstract

**Background:**

Biliary cancer includes cancer of the gallbladder as well as extrahepatic and intrahepatic cholangiocarcinoma. Surgery is the only curative treatment option available. Recently, much more aggressive surgical approaches have been employed. Therefore, we have investigated outcome of biliary cancer before and after establishment of an aggressive surgical approach.

**Methods:**

Retrospective single-center analysis comparing two time periods of 5 years each. During the second period new surgical expertise and a much more aggressive surgical approach were used.

**Results:**

In the first time period (5/1995–4/2000) only 29 patients with biliary cancer were treated at our institution, while a total of 85 patients were treated during the second time period (5/2000–4/2005). Surgical resection was attempted in 55% during the first period versus 62% in the second; resection was complete in 37.5% and 58.5%, respectively. Patients undergoing resection during the second time period were more likely to be without relapse compared with patients undergoing resection in the first time period. No patient from the first period is without evidence of disease, compared to 11 patients operated in the second period. Resected patients had better survival compared with unresected patients for all tumor locations (gallbladder, extrahepatic and intrahepatic cholangiocarcinomas). Overall survival of patients was not significantly different between patients treated during the first versus the second time period.

**Conclusion:**

In patients with biliary cancer surgical resection should be attempted whenever possible. However, long-term survival can be achieved only when a complete resection is obtained.

## Background

Biliary cancer is a rare disease with a bad prognosis. This disease encompasses gallbladder cancer, extrahepatic cholangiocarcinoma of the perihilar region and of the distal choledochus as well as intrahepatic cholangiocarcinoma. (The latter location was traditionally grouped with liver tumours). In recent years more active surgical and chemotherapeutic approaches in the treatment of biliary cancer have been employed. The impact of these approaches is difficult to assess although based on surgical series[1,2] and a single randomized trial of chemotherapy versus best supportive care[3] these treatments have become general practice. In this retrospective single center series we analyse the impact on outcome of the increasing use of an aggressive surgical approach.

## Methods

We have performed a retrospective analysis of patients with biliary cancer treated at the University Hospital in Zurich over a 10 year period. Patients who had part of their treatment at other institutions were not excluded. Patients were seen in the departments of visceral surgery (43), oncology (41), both of these (19), or gastroenterology (11). In all cases with missing follow-up information, a letter with paid feed-back envelope was mailed to physicians and/or other hospitals where patients were treated to obtain a maximum of information.

In May 2000 a new head of visceral surgery was elected with particular interest and expertise in biliary and liver surgery. Since that time a much more active surgical approach is used in the treatment of biliary cancer at our institution. We were interested to analyse the impact of this change in approach on patient outcome. Therefore, we divided the patient population into two time periods of equal duration based on the date of diagnosis. The first time period includes patients with a diagnosis of biliary cancer made between May 1, 1995 and April 30, 2000. The second time period includes patients with a diagnosis made between May 1, 2000 and April 30, 2005. Patient and tumor characteristics, diagnostic and therapeutic procedures including type of surgery or biopsy, pathology, imaging (including positron-emission tomography since 2001 to exclude metastatic disease),[4] laboratory findings, chemotherapy, radiotherapy, and outcome were tabulated and analysed by descriptive statistics. P-values were calculated with log-rank test.

## Results

### Patient and tumor characteristics

Patient characteristics and tumor types are listed in Table 1. In the first five-year period (May 1, 1995 until April 30, 2000) only 29 patients with a diagnosis of biliary cancer were treated at our institution. By contrast, in the second five-year period (May 1, 2000 until April 30, 2005) 85 patients were seen. Median age was similar, 60 (range 32–84) and 63 (34–80), respectively. There were 55.2% females in the first period, and 51.8% in the second period. Remarkably, in the first time period the proportion of gallbladder cancer was much higher (44.8%) than in the recent period (25.9%). Similarly, the proportion of ductal cholangiocarcinomas was much lower in the first time period, especially the intrahepatic cholangiocarcinomas: 13.8% in the first time period versus 27.1% in the recent past. The proportion of extrahepatic cholangiocarcinomas was 37.9% and 45.9%, respectively.

**Table 1:** Patient characteristics and tumor type

Characteristic	1995–2000		2000–2005	
	N	%	N	%

Number of patients	29	100	85	100
Median age (range)	60 (32–84)		63 (34–80)	
Gender female/male	16/13	55.2/44.8	44/41	51.8/48.2
Gallbladder carcinoma	13	44.8	22	25.9
Intrahepatic CCC	4	13.8	23	27.1
Extrahepatic CCC	11	37.9	39	45.9
Tumor localization unknown	1	3.4	1	1.2

CCC = cholangiocarcinoma

Diagnostic procedures, type of surgery, and surgical pathology are listed in Table 2. Diagnosis was most often made by tumor biopsy or tumor resection, and only rarely by cytology. Type of surgery has significantly changed in the second time period with 44.7% liver resections with curative intent compared to only 17.2% in the first time period. Conversely, smaller resections like resection of extrahepatic bile ducts alone (10.3% versus 4.7%) or cholecystectomy only (17.2 % versus 4.7%) as well as surgery without resection (17.2% versus 14.1%) were more frequently performed in the first time period. Not surprisingly, histology was adenocarcinoma in a very large majority, with a few cases of papillary adenocarcinoma and squamous cell carcinoma in the second time period. Tumor grading was similar in the two time periods; grading in the first/the second time period: G1 10.3%/5.9%, G2 34.5%/52.9%, G3 31.0%/16.5%, unknown 24.1%/24.7%.

**Table 2:** Diagnostic procedures, type of surgery, surgical pathology

Time period	1995–2000		2000–2005	
	N	%	N	%

Number of patients	29	100	85	100
**DIAGNOSTIC PROCEDURE**				
Resection specimen	10	34.5	42	50.0
Tumor biopsy	17	58.6	38	45.2
Brush cytology	2	6.9	3	3.6
Diagnosis established by follow-up	0	-	1	1.2
**SURGERY**				
**Resection**	**16**	**55.2**	**53**	**62.4**
Liver resection	5	17.2	38	44.7
Whipple operation	2	6.9	6	7.1
Transplantation	1	3.4	1	1.2
Resection of extrahepatic bile ducts only	3	10.3	4	4.7
Cholecystectomy only	5	17.2	4	4.7
**Non-resection surgery**	**5**	**17.2**	**12**	**14.1**
Excision of lymph nodes only	0	-	1	1.2
Exploratory laparoscopy	1	3.4	6	7.1
Exploratory laparotomy	2	6.9	4	4.7
Surgical biopsy	2	6.9	1	1.2
**No surgery**	**8**	**27.6**	**20**	**23.5**
**SURGICAL PATHOLOGY**				
Adenocarcinoma NOS	26	89.7	75	88.2
Papillary adenocarcinoma	0	-	3	3.5
Squamous cell carcinoma	0	-	3	3.5
Unknown	3	10.3	4	4.7

NOS = not otherwise specified

TNM-staging of all patients is shown in Table 3. It is evident that a correct T-stage could not be assigned based on imaging alone, but only when a resection was performed. Conversely, in about one third of patients, assignment of the N-stage was based on imaging studies. CT-scanning was performed in most patients: 84.0% in the first time period, and 87.2% in the second. Use of ultrasound has decreased, from 80% in the first time period to 57.7% in the second. On the contrary, the use of MRI has increased from 16.0% to 34.6%, respectively. Use of the tumor marker CA 19-9 has also increased from 27.6% to 63.5%. The preoperative values measured are very similar: Median (range) CA 19-9 was 72.8 kU/l (4.9–1340) in the first time period, compared with 74.8 kU/l (0-72'900) in the second time period.

**Table 3:** Staging

**Time period**	1995–2000		2000–2005	
**Resection**	**Yes**	**No**	**Yes**	**No**

Number of patients	16	13	53	32
**T-Stage**				
T1	0	0	11	0
T2	5	0	11	0
T3	5	0	27	0
T4	3	0	3	0
Unknown	3	13	1	32
**N-Stage**				
N0	4	2	31	3
N1	6	2	17	8
Unknown	6	9	5	21
**M-Stage**				
M0	6	1	46	13
M1	2	7	4	12
Unknown	8	5	3	7

### Treatment

**Figure 1:**
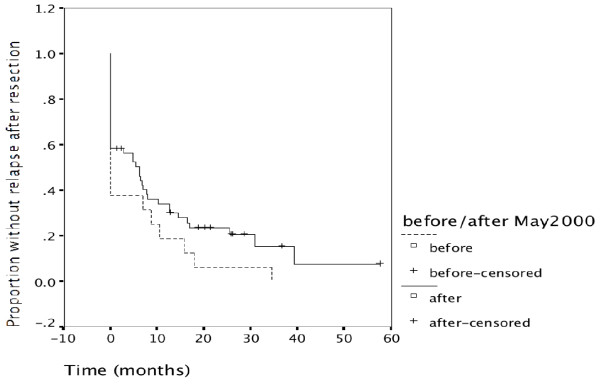
**Proportion without relapse after resection**. The proportion of patients without relapse over time is shown for the two time periods. In the first period only 6 of 16 resected patients had complete resections (37.5%), 10 have relapsed immediately. In the second time period 31 of 53 patients had complete resections (58.5%), 22 had relapsed immediately. Similarly during further follow-up, time to relapse after resection remained increased for the second time period (p = 0.064).

The only curative treatment of cholangiocarcinoma is surgical resection. In the first time period, 12 of 29 patients underwent resection with curative intent (41.4%), compared to 51 of 85 (60.0%) in the second time period. Follow-up of surgically resected patients is shown in Figure 1. Time to relapse is longer for patients treated in the second time period (p = 0.064). In the first time period only 6 of 16 (37.5%) resections were complete resections, compared to 31 of 53 (58.5%) resections in the second time period. All 6 patients of the first time period having undergone complete resection have relapsed. So far, 20 of the 31 later patients having undergone complete resection have relapsed, while 11 patients are without evidence of disease (2 of them lost to follow-up). One exceptional case has been successfully treated with combined modality therapy involving neoadjuvant chemo-radiation followed by living donor liver transplantation from a sibling who had previously donated bone marrow to the patient for acute lymphoblastic leukaemia.[5,6]

Chemotherapy was used in about half the patients: 44.8% in the first time period, and 55.3% in the second period. Two patients received adjuvant chemotherapy, while in all other patients chemotherapy was given with palliative intent. In early times fluorouracil (23.8%), gemcitabine (28.6%) or the ECF (epirubicin-cisplatin-fluorouracil) combination (28.6%) were applied while in the second period gemcitabine monotherapy was the dominant choice (58%), followed by ECF (16.1%). There was no significant survival difference in the 13 patients treated with chemotherapy during the first time period compared to the 47 patients treated with chemotherapy during the second time period. In addition, overall survival was similar from diagnosis between all patients undergoing chemotherapy at some point in time compared to all patients never receiving chemotherapy.

Radiation treatment was given with neoadjuvant intent to 1 exceptional patient (see above), to 2 patients with adjuvant intent, and to 10 patients with palliative intent (1 to primary tumor, 9 to metastases).

### Survival

**Figure 2:**
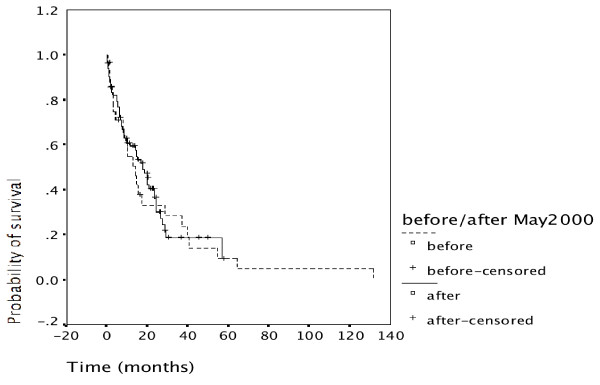
**Overall survival**. Overall survival of patients treated in the first time period is comparable to the second time period. Survival of gallbladder carcinoma as well as of cholangiocarcinoma (intrahepatic and extrahepatic) was also similar during the first and the second time period (not shown).

Overall survival is shown in Figure 2. Patients treated in the first time period had a similar survival from diagnosis as patients in the second time period. Similarly, when analysing survival according to primary tumor location (see Table 1), no significant differences in overall survival were found. Comparison was made in 13 versus 22 carcinomas of the gallbladder, 4 versus 23 intrahepatic cholangiocarcinomas, and 11 versus 39 extrahepatic cholangiocarcinomas (figures not shown).

**Figure 3:**
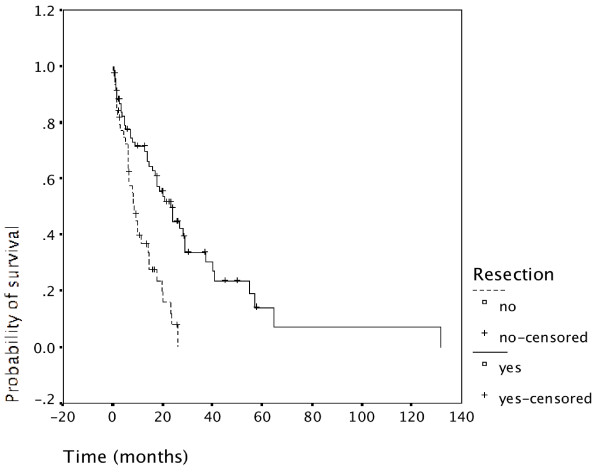
**Resection and survival**. Survival of patients undergoing resection was significantly increased compared with patients not undergoing resection (p = 0.000). This is shown for all patients. It was also true for patients of the first time period (N = 29), and for patients of the second time period (N = 85).

Overall survival in resected patients is significantly longer than in non-resected patients (p = 0.000) as shown in Figure 3. This was true and very alike for both time periods (data not shown). Taking a close look at Figure 3 shows that no un-resected patient has survived 3 years, while more than 30% of resected patients are alive at the 3-year time interval.

**Figure 4:**
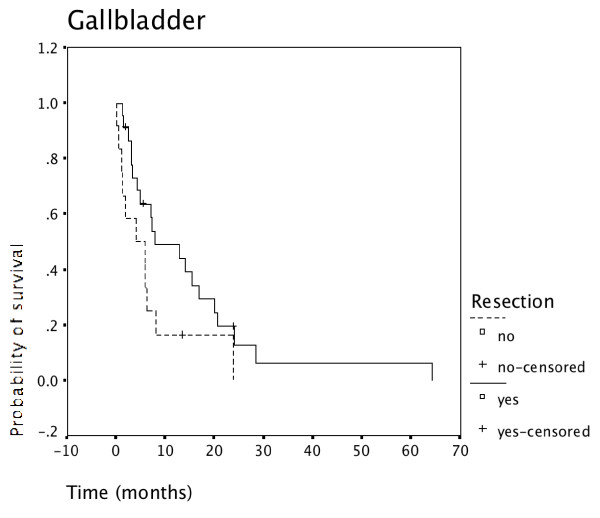
**Resection for gallbladder cancer and survival**. Survival of patients having undergone resection for gallbladder cancer was superior to survival of non-resected patients (N = 35).

**Figure 5:**
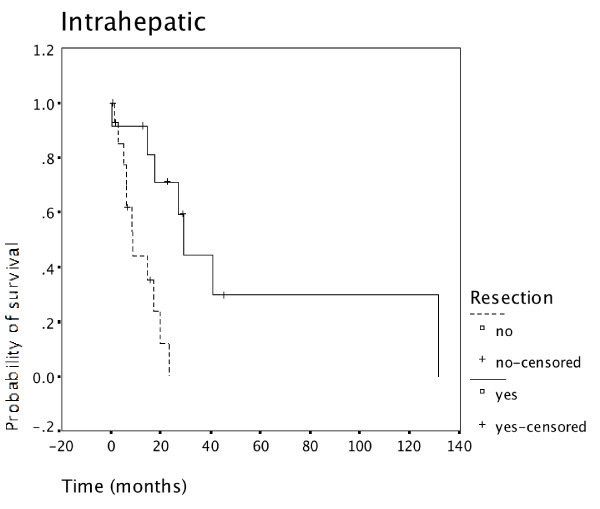
**Resection for intrahepatic cholangiocarcinoma and survival**. Survival of patients having undergone resection for intrahepatic cholangiocarcinoma was superior to survival of non-resected patients (N = 27).

**Figure 6:**
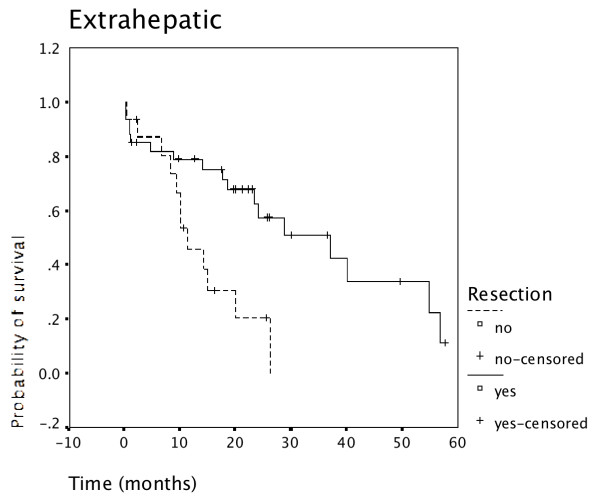
**Resection for extrahepatic cholangiocarcinoma and survival**. Survival of patients having undergone resection for extrahepatic cholangiocarcinoma was superior to survival of non-resected patients (N = 50).

Resected patients had superior survival to non-resected patients in all 3 tumor locations: Carcinoma of the gallbladder (Figure 4), intrahepatic cholangiocarcinoma (Figure 5), as well as extrahepatic cholangiocarcinoma (Figure 6).

**Figure 7:**
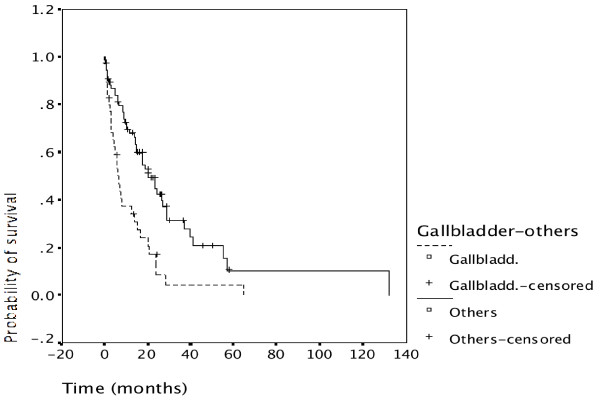
**Survival and tumor location**. Survival was related to tumor location. Gallbladder carcinoma had significantly worse prognosis than cholangiocarcinoma (p = 0.001). There was no significant difference between intrahepatic cholangiocarcinoma (ICC) and extrahepatic cholangiocarcinoma (ECC) while both had significantly better survival than gallbladder carcinoma. Median survival of gallbladder cancer was about 8 months, for ICC 20 months, for ECC 22 months.

Survival in carcinoma of the gallbladder was significantly worse than survival in ductal cholangiocarcinoma (p = 0.001) as shown in Figure 7. This was also true when either intrahepatic or extrahepatic cholangiocarcinoma was compared separately with gallbladder carcinoma. Survival of intrahepatic versus extrahepatic cholangiocarcinoma was not significantly different with a minimal advantage for extrahepatic disease (data not shown).

Table 4 shows outcome, and how it relates to initial stage. It is apparent that the only patients free of disease are those who have a T1, T2, or rarely T3 disease. There was not a single patient with lymph node involvement who has not relapsed.

**Table 4:** Outcome

**Disease status**	NED	AWD	DOD
**T-Stage**			
T1	6	1	4
T2	4	2	10
T3	1	9	22
T4	0	1	5
Unknown	0	11	38
**N-Stage**			
N0	11	7	22
N1	0	8	25
Unknown	0	9	32
**M-Stage**			
M0	11	14	41
M1	0	6	19
Unknown	0	4	19

NED = No evidence of disease. AWD = Alive with disease. DOD = Dead of disease

## Discussion

In this retrospective study we have compared patients with biliary cancer treated at our institution in two time periods. With the advent of new expertise in the department of visceral surgery the patient volume with biliary cancer treated at our institution has almost trippled in the second period, increasing from less than 6 patients per year to 17 patients per year. This is certainly due to a change in referral, and not due to an increase in the incidence of the disease nor to changes in diagnostic evaluation. It is very likely that many patients with biliary cancer diagnosed during the first period (5/1995–4/2000) were simply not referred, probably for various reasons including the perception that therapeutic options were limited. It is likely that this has lead to "backward stage migration": While only the "best" patients were operated during the first period, patients with more advanced disease were referred during the second time period. In the first period, almost half the patients had gallbladder tumors; some of them had incidental gallbladder adenocarcinoma diagnosed histologically after cholecystectomy. In the second period the proportion of intrahepatic and extrahepatic cholangiocarcinomas necessitating more extensive and more demanding surgery was much higher.

Despite this referral bias the proportion of patients undergoing resection was slightly higher during the second time period (62%, Table 2) than the first time period (55%). Patients undergoing resection had a significantly better outcome than non-resected patients (p = 0.000) (Figure 3). The advantage of undergoing resection was more marked for extrahepatic cholangiocarcinoma than for intrahepatic cholangiocarcinoma and gallbladder cancer (Figure 4, 5, 6). In addition, during the second time period resection was more often complete and remission (time without relapse) was prolonged (Figure 1). The only patients without evidence of disease at this time are patients having undergone resection for a low T-stage (T1, T2 and very rarely T3) and N-stage (N0 only) (Table 4). In the literature, the prognostic importance of N-stage and the worth of extensive lymph node dissections is controversial.[1,7,8] Most authors favour an aggressive and extensive surgical approach. Unfortunately, in this analysis probably skewed by referral bias, we were unable to document increased overall survival for patients treated during the second time period (Figure 2). There are many explanations possible for this lack of better survival. The populations of the two time periods are dissimilar in many respects besides surgery, including type and proportion of patients referred, location of the primary tumor, stage distribution, diagnostic evaluations, palliative treatments.

In the second time period the use of chemotherapy has increased somewhat, from 45% to 55%. Chemotherapy during the first time period was mostly fluorouracil-based (57%) while during the second time period gemcitabine-based chemotherapy (64%) was used most frequently. We were unable to find a difference in overall survival between patients treated with chemotherapy during the second time period when compared with the first time period. Looking at the variety of regimes and the differences in the patient populations as well as their restricted numbers (13 patients in the first time period, 47 in the second) no conclusions about palliative chemotherapy in biliary cancer can be drawn. Similarly, in the literature the worth of chemotherapy is controversial.[9] Nevertheless, gemcitabine with or without capecitabine is considered a reasonable choice for palliative treatment of advanced biliary cancer.[10]

In conclusion, for the treatment of biliary cancer it is reasonable to attempt complete resection, while incomplete resections are probably of no avail.[7,11-13] We will follow the 11 patients without evidence of disease from the second time period in order to define how many (if any) of them have been cured. No patient from the first time period is without relapse. In order to define the worth of chemotherapy in adjvanced biliary cancer a randomized multicenter study comparing gemcitabine with the combination of gemcitabine/capecitabine is planned.[10]

## Competing interests

NR, AK, and BCP have neither financial nor non-financial competing interests with this publication.

## Authors' contributions

NR performed the acquisition of the data, participated in the analysis and interpretation of the data and drafting of the manuscript, AK conceived of the study and helped with its design, BCP developed the design, planned the data acquisition, performed their analysis and interpretation, and drafted the manuscript. All authors read and approved the final manuscript.
